# Effect of daptomycin and vancomycin on *Staphylococcus epidermidis* biofilms: An *in vitro* assessment using fluorescence *in situ* hybridization

**DOI:** 10.1371/journal.pone.0221786

**Published:** 2019-08-27

**Authors:** S. Sutrave, J. Kikhney, J. Schmidt, A. Petrich, A. Wiessner, Laura Kursawe, M. Gebhardt, U. Kertzscher, G. Gabel, L. Goubergrits, K. Affeld, A. Moter

**Affiliations:** 1 Biofilmcenter, Institute for Microbiology, Infectious Diseases and Immunology, Charité – Universitätsmedizin Berlin, corporate member of Freie Universität Berlin, Humboldt-Universität zu Berlin, and Berlin Institute of Health, Berlin, Germany; 2 Biofluid Mechanics Laboratory, Charité – Universitätsmedizin Berlin, corporate member of Freie Universität Berlin, Humboldt-Universität zu Berlin, and Berlin Institute of Health, Berlin, Germany; Leibniz-Institute DSMZ, GERMANY

## Abstract

Colonization of in-dwelling catheters by microbial biofilms is a major concern in patient health eventually leading to catheter-related blood stream infections. Biofilms are less susceptible to standard antibiotic therapies that are effective against planktonic bacteria. Standard procedure for the detection of microorganisms on the catheter tip is culture. However, viable but non-culturable cells (VBNCs) may be missed. The aim of this study was to evaluate the use of fluorescence *in situ* hybridization (FISH) as an indicator to visualize and quantify the effect of the antibiotics daptomycin and vancomycin on biofilms *in situ*. We established an *in vitro* catheter biofilm model of *Staphylococcus epidermidis* biofilms on polyurethane catheters. Biofilm activity was measured by FISH and correlated to colony forming units (CFU) data. Digital image analysis was used for quantification of total biofilm mass and the area of the FISH positive biofilm cells. FISH showed a pronounced effect of both antibiotics on the biofilms, with daptomycin having a significantly stronger effect in terms of both reduction of biofilm mass and number of FISH-positive cells. This supports the anti-biofilm capacity of daptomycin. Interestingly, neither antibiotic was able to eradicate all of the FISH-positive cells. In summary, FISH succeeded in visualization, quantification, and localization of antibiotic activity on biofilms. This technique adds a new tool to the arsenal of test systems for anti-biofilm compounds. FISH is a valuable complementary technique to CFU since it can be highly standardized and provides information on biofilm architecture and quantity and localization of survivor cells.

## Introduction

In-dwelling medical devices such as central-venous catheters can become colonized by biofilms, leading to severe bacteraemia and sepsis. Biofilm-associated infections are tolerant to standard antibiotic regimens making treatment difficult [1]. Catheter-related blood stream infections have been shown to increase the financial burden for intensive care unit patients and the health care system in addition to causing higher patient mortality [2]. Long-term use of central venous catheters is associated with a risk of bloodstream infection and sepsis (2.7 cases per 1000 catheter-days) [3].

Vancomycin is the standard antibiotic agent for empirical therapy in cases of catheter-related blood stream infections due to methicillin-resistant *Staphylococcus* spp.; nonetheless, for vancomycin minimal inhibitory concentrations (MICs) >2 μg/mL alternative treatment with daptomycin is recommended [4]. Emergence of resistance to vancomycin in blood stream infection cases of methicillin-resistant coagulase negative staphylococci caused by higher MICs has previously been reported [5, 6].

Newer antibiotics such as daptomycin are in use for the strains that are difficult to eradicate. Since 2006 daptomycin has been in use against bacteraemia and right-sided endocarditis caused by *S*. *aureus* [7]. Several studies have compared the efficacy of daptomycin to that of other antibiotics and concluded that daptomycin has potent activity in the treatment of staphylococcal biofilm-related infections [8, 9].

According to the manufacturer, daptomycin has a distinct mode of action in Gram-positive bacteria. In a calcium-dependent binding, daptomycin causes depolarization of the cell membrane followed by a shutdown of cellular processes such as nucleic acid and protein synthesis. The patent for daptomycin expired in 2016 and generic forms are now available for use.

Previously, biofilm disinfection studies examining the effect of antimicrobials on biofilms have used disintegration of the biofilm and count of colony forming units (CFU) as a measure of antimicrobial efficacy [10]. CFU analysis gives an incomplete picture of cell viability in biofilms following antibiotic treatment. The development of persister cells, which are not detected by CFU measurements, causes recurrent infection, making antibiotic treatment even more challenging [11].

16S rRNA directed fluorescence *in situ* hybridization (FISH) is a culture-independent, tool for visualization and identification of bacteria. The intensity of the FISH signal directly correlates to the ribosomal content within the cells and is therefore an indirect indicator of activity. The objective of this study was to combine digital image analysis and FISH to visualize, precisely locate and quantify the activity of antibiotics on *Staphylococcus epidermidis* biofilms formed in vitro.

## Materials and methods

### In vitro model for biofilm formation on catheters

The bacterial test strain chosen for this study is the polysaccharide intercellular adhesin (PIA) positive *S*. *epidermidis* strain 8400 isolated in 1992 from a clinical sample [12]. This strain is well characterized and has been shown to build mature and reproducible biofilms. Olson et al. showed that PIA-dependent *S*. *epidermidis* biofilms have a higher tolerance to antibiotics than their PIA-independent counterparts [13]. Biofilms were grown in 50 ml-scale biofilm reactors (see S1 Fig). 10% tryptic soy broth (TSB) was inoculated with the clinical isolate *S*. *epidermidis* strain 8400, an optical density (600nm) of 0.3 of the resulting suspension was achieved and the suspension was incubated with the catheters at 37°C for 7 days. The medium was replaced with fresh 100% TSB medium supplemented with 25% glucose every 24 hours. The catheters remained immersed in the medium throughout the experiment while being continuously agitated by a magnetic stirrer. During this time a biofilm formed in the lumen and on the outside of the polyurethane catheters.

### Antimicrobial treatment

The concentrations of daptomycin (Novartis Pharma AG, Basel, Switzerland) of 160 μg/mL and vancomycin (Sigma-Aldrich Chemie GmbH, Munich, Germany) 100 μg/mL were chosen according to the described peak plasma concentrations in humans [14, 15]. The TSB medium was replaced with Mueller-Hinton medium supplemented with calcium along with daptomycin or vancomycin at the above concentrations, and pumped (50 μl/min) through the catheters into the bioreactor. Calcium was added to the medium due to the calcium-dependent mode of action of daptomycin. The control reactor was treated accordingly with medium supplemented with calcium and phosphate buffered saline (PBS). The catheters remained for 24 h at 37°C in the antibiotic/control solution which was being stirred continuously to ensure a homogeneous mixture of antibiotics in the medium.

The catheters were harvested and cut into two halves. One half (1 cm) of each catheter was used for CFU analysis; the other half was used for FISH analysis.

### CFU measurement

The section of the catheter to be used for CFU analysis was transferred to 1 ml PBS (pH 7.4) and vortexed for 1 minute to homogenize the biofilm. Serial dilution was carried out, 100 μl aliquots were plated on Muller-Hinton Agar and the plates were incubated for 48 h at 37°C. Colonies were then counted and final counts were calculated taking the dilution factor into account.

### Sample preparation and FISH

One half of each catheter was prepared for FISH by fixation and embedding as described [16]. Each catheter was cut into four pieces; the pieces were then embedded upright in cold polymerizing resin Technovit 8100 (Kulzer, Wehrheim, Germany). Using a microtome, 2μm thick sections of the catheter pieces were cut into 8 cross-sections with a total of 32 cross-sections per catheter (see S2 Fig).

Enzymatic pretreatment using lysozyme and lysostaphin was conducted as described [17]. Control slides with cultures of *Escherichia coli*, *S*. *aureus* and *Streptococcus pyogenes* were included in every experiment.

The hybridization buffer containing the nucleic acid stain 4′,6-diamidino-2-phenylindole (DAPI) and the oligonucleotide probes EUB338 (specific for most bacterial species), STAPHY specific for *Staphylococcus* spp., and non-EUB338 to rule out unspecific probe binding was applied to the sections [17–19]. EUB338 and STAPHY were labelled at the 5’ end with the fluorescent indocarbocyanine dye Cy3; non-EUB338 was labelled with Cy5. Hybridization, washing and mounting was carried out as described [17].

### Epifluorescence microscopy and digital image analysis

Epifluorescence microscopy was carried out as previously published [20]. Images were obtained with the help of AxioCam MRm (Zeiss) using the AxioVision 4.6 software.

A total of 47 catheters was investigated: control (n = 24), vancomycin (n = 11) and daptomycin (n = 12). For each catheter 100x magnification images of 32 cross-sections at different planes were taken, each with two images of the outer surface of the catheter, resulting in a total of 64 images per catheter. Thus, the total number of images statistically evaluated was as follows: control (n = 1536), vancomycin (n = 704) and daptomycin (n = 768).

Quantification of biofilm area and percentage of FISH-positive cells was achieved using the Adobe After Effects 5.5 software and the image analysis program *daime* [21]. The DAPI and Cy3 grayscale images were transformed into binary images using the luminance threshold setting option of “after effects” and exported as previously published [20]. The images were then segmented using *daime* and artefacts were removed where necessary. The *daime* program was employed to calculate the biofilm areas of both the Cy3 and the DAPI channels with the DAPI area set as mask for the Cy3 layer. The total biofilm area per catheter (DAPI) and the percentage of the FISH-positive fraction (Cy3) were thus calculated.

### Statistical analysis

The data was analysed using the statistical package SPSS V.19 (IBM, USA). Significance was assumed at p≤0.05 for all tests. A Kolmogorov-Smirnov test was performed to assess deviations from a normal distribution. Group differences were assessed by paired Student’s t-test in the case of normally distributed data; otherwise the Mann-Whitney U test was used in combination with Levene’s test to prove the equality of variances.

## Results

### In vitro catheter biofilm model

The *in vitro* catheter biofilm model successfully produced thick biofilms on the outer surface of the catheters. The biofilm formation within the catheter lumen was less pronounced, ranging widely from a few cells to dense biofilms. The *S*. *epidermidis* PIA 8400 strain chosen for the present study showed variation in the amount of biofilm obtained, in spite of the same stringent experimental conditions being maintained.

### FISH and digital image analysis

The EUB338-Cy3 and STAPHY-Cy3 FISH probes hybridized successfully to the biofilm samples showing bright orange fluorescence. No unspecific probe binding was detected with the non-EUB338-Cy5 probe. DAPI stained nucleic acids were observed emitting blue fluorescence. Characteristic biofilm was observed in the biofilms obtained from the *in vitro* model. A distinct pattern of distribution of the FISH-positive cells was seen; the FISH-positive cells were densely clustered towards the periphery of the biofilm, with a few single cells found scattered deeper within the biofilm.

**Fig 1:**
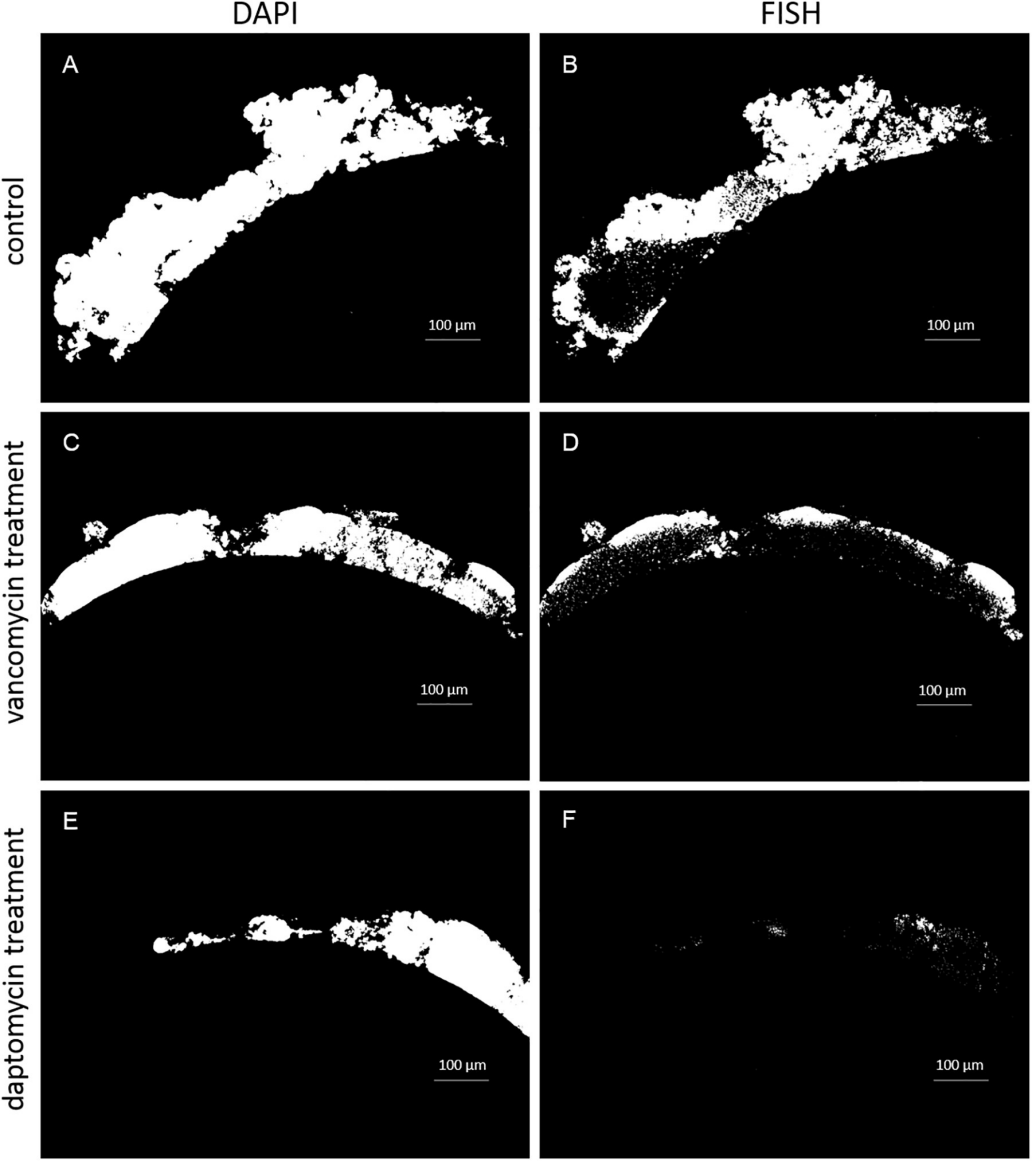
Digital image analysis using *daime* shows the reduction of total biofilm area and FISH-positive fraction, respectively, for *in vitro S*. *epidermidis*. A and B show DAPI and FISH masks, respectively, of the control biofilm treated with PBS. C and D show DAPI and FISH masks for biofilm on a catheter treated with vancomycin, and E and F show DAPI and FISH masks for biofilm on a catheter treated with daptomycin, respectively. The reduction of FISH positive areas after treatment with vancomycin (D) and even more with daptomycin (F) is clearly visible.

A major reduction in the total area of biofilm on catheters treated with daptomycin and vancomycin as compared to the controls was seen as well. Daptomycin showed a greater reduction in total biofilm area than vancomycin, establishing a trend mirrored in the reduction of the FISH-positive fraction (Fig 1). However, due to a high standard deviation this difference was not statistically significant at p≤0.05.

**Fig 2:**
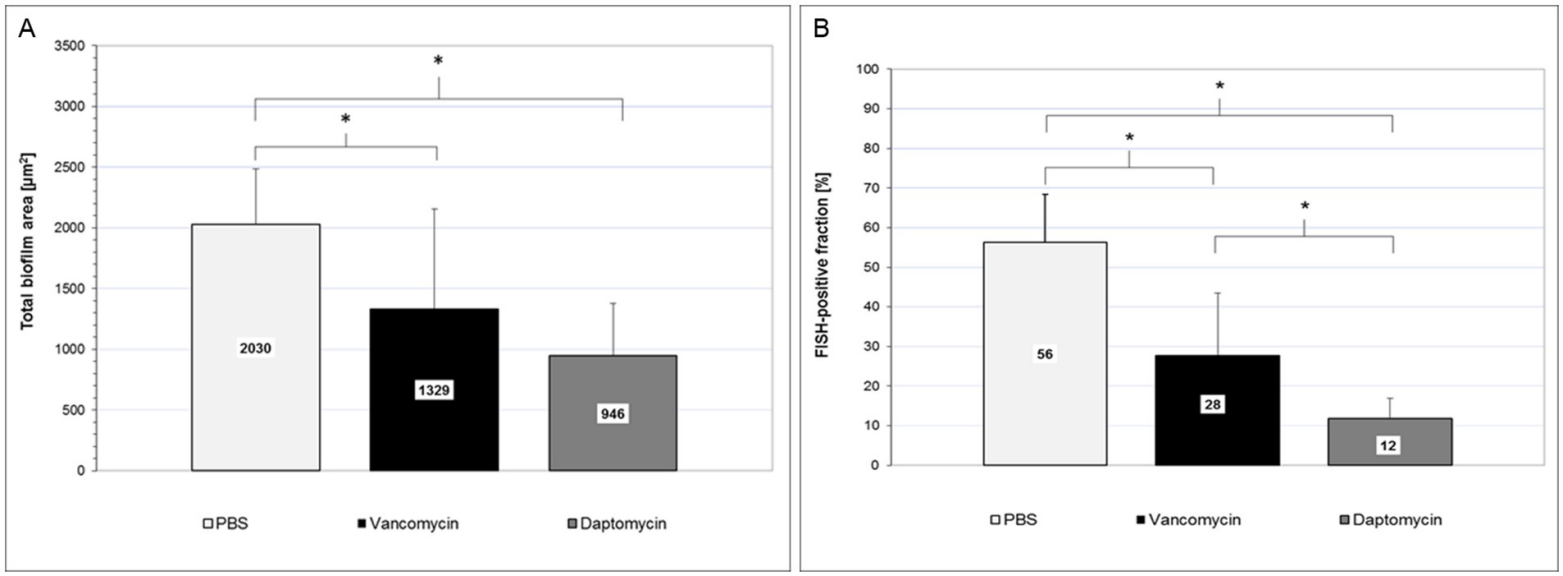
Reduction of the total biofilm mass (A) and FISH-positive fraction (B) in biofilms under antibiotic treatment. Total biofilm area [μm^2^] represented by the DAPI mask in *daime* and fraction of FISH-positive cells in [%] as compared to the total biofilm area, for *in vitro* grown biofilms of *S*. *epidermidis* treated with PBS, vancomycin and daptomycin, respectively. Data was found to be normally distributed; therefore, mean values and Student’s t-test were used to denote group differences. *significant difference between test groups at p≤0.05.

The percentage of FISH-positive cells was significantly reduced within the biofilm area evaluated. Both antibiotics reduced total biofilm area; notably, daptomycin showed a greater reduction in FISH-positive cells than vancomycin compared to the control. Following treatment with PBS (control) and antibiotics, the percentage of FISH-positive cells was found to be 56% for the control, but reduced to 28% after vancomycin and to 12% after daptomycin treatment, respectively (Fig 2).

**Fig 3:**
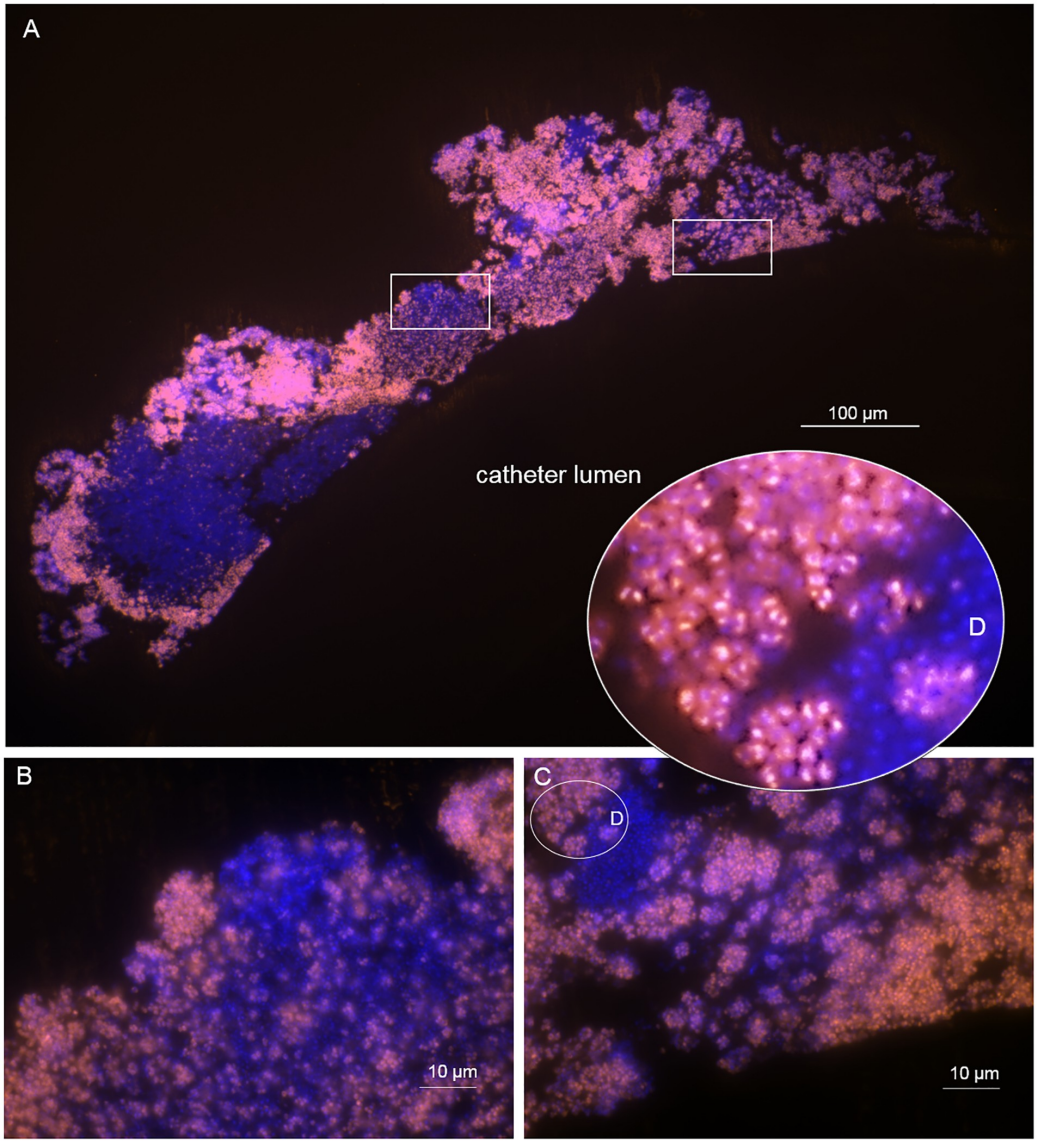
High proportion of FISH-positive cells in biofilms on a control catheter. *In vitro S*. *epidermidis* biofilm on control catheter treated with PBS. The blue layer represents the nucleic acid stain DAPI. The orange layer represents the FISH-positive cells (Cy3). B, C are magnifications of the insets from A showing FISH-positive cocci. At higher magnification of inset D, single bacterial cells are visible with differential FISH signal intensity. Of interest, note the bright fluorescence in particular in double-cocci that seem to divide, whereas the blue cocci resemble cells with low ribosome content.

The control biofilms, treated with PBS, showed a distinct pattern of distribution of the FISH-positive cells within the biofilms (Fig 3). The outer periphery of the biofilm showed a bright FISH-positive signal along with scattered cells found towards the centre of the biofilm mass and no FISH-positive cells on the areas of the biofilm adjacent to the catheter.

**Fig 4:**
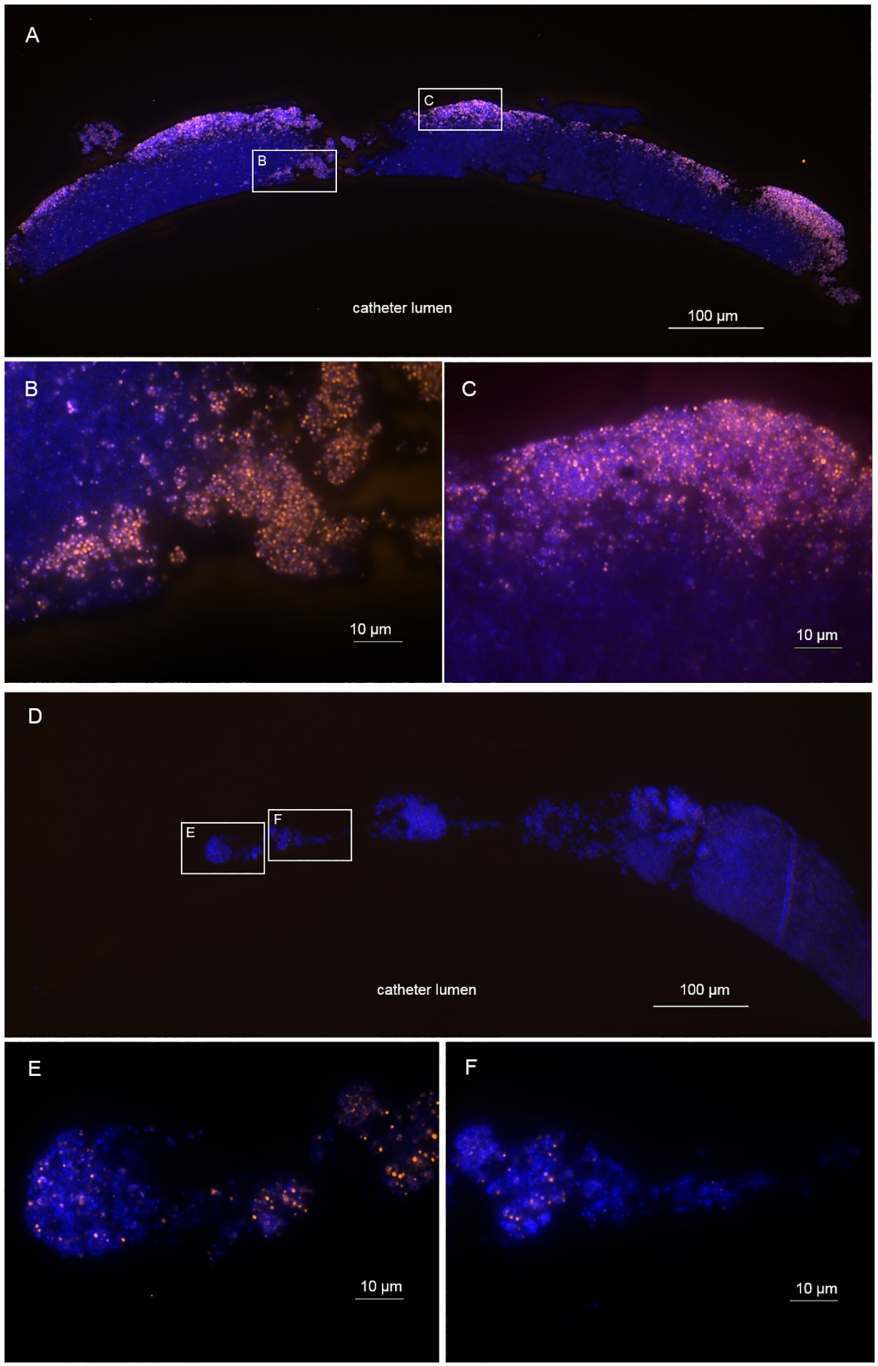
FISH shows differential pattern of remaining FISH-positive bacteria in biofilms and reduction of the FISH-positive fraction upon antibiotic treatment. *In vitro S*. *epidermidis* biofilm on catheter treated with vancomycin (4A-C) or daptomycin (4D-F), respectively. The blue layer represents the nucleic acid stain DAPI. The orange layer represents the FISH positive, ribosome-rich cells (Cy3). B, C are magnifications of the insets from A showing FISH-positive cocci in the outer layers of the vancomycin treated biofilm. Upon daptomycin treatment (4D-F), only single cells remain FISH-positive in all parts of the biofilm. 4E and F are magnifications of the insets from 4D showing single FISH-positive cocci remaining under daptomycin treatment.

Biofilms treated with vancomycin exhibited the same distribution of FISH-positive cells as seen in the case of the controls, there being a higher density of these cells towards the outer edges of the biofilm (Fig 4A–4C). Daptomycin treated biofilms showed single FISH-positive cells spread throughout the entire mass of the biofilm (Fig 4D–4F).

### FISH vs. CFU data

Log_10_ CFU/mL values for control catheters and catheters treated with vancomycin and daptomycin were 12.15±0.82, 4.91±0.28 and 2.49±0.57, respectively. Statistical differences between any test groups (control, vancomycin and daptomycin) were highly significant (p≤0.01). Vancomycin and daptomycin met and exceeded the 3-log reduction in CFU/mL criteria to be considered bactericidal [22]. As evidenced by the cells that are FISH-positive after antibiotic treatment, neither antibiotic achieved a 100 percent kill in the *in vitro* experimental setup described here. The CFU data, as expected, showed the same general pattern of reduction in cell count following the application of antibiotics as FISH, but without the spatial aspect of distribution of cells within the biofilm gained via FISH.

## Discussion

The validity of FISH in establishing the improved efficacy of antibiotic-loaded microparticles in staphylococcal biofilms has recently been shown [23]. The present study demonstrates, for the first time, the use of FISH for visualization, quantification, and localization of the rRNA containing cells within a catheter-related biofilm following antibiotic treatment. Daptomycin reduced the total biofilm area as well as the percentage of FISH-positive cells of the *in vitro S*. *epidermidis* biofilms; vancomycin showed this reduction to a lesser extent. FISH demonstrated distinct differences in the distribution of FISH-positive cells between the two antibiotics, perhaps due to their disparate modes of action. Our results prove FISH to be an invaluable addition to the current arsenal of tools available for the evaluation of antibiotic action on biofilms.

As expected, the FISH data correlate to the CFU results. It is important to point out the advantages of FISH over CFU towards a better understanding of biofilms under antibiotic therapy. Firstly, FISH provides spatial resolution aiding in visual input and localization within the biofilm. Secondly, FISH enables quantification of the biofilm in terms of area and percentage of FISH-positive cells. Lastly, FISH helps us examine and interpret the means by which the surviving cells lead to clinical relapse as well as provide a feeding ground for new and incoming bacterial cells.

Various *in vitro* models of biofilm formation for testing antimicrobial activity have been published so far [13, 24, 25]. In the current stage of research on catheter-related infections, there is a lack of standardized models for testing *in vitro* biofilms on catheter surfaces [26]. The *in vitro* model developed in this study produced thick biofilms on the catheters, but the biofilm growth was not uniform between catheters within each biofilm reactor. Furthermore, the long turnaround time per experiment limits the use of the model as a prototype for *in vitro* biofilm studies on catheters. A shorter incubation time for biofilm growths such as 6–10 hours could reduce the standard deviation and speed up the analysis. In this study we aimed at rich biofilms to demonstrate the feasibility of analysis of biofilm architecture to be able to compare the anti-biofilm activity of two antibiotics. Furthermore, as a clinical situation we aimed at being correspondent to port systems.

An *in vitro* model of *S*. *epidermidis* device-related infections for antibiotic testing has been shown to be a valid first step before moving to an in vivo model [27]. The standard protocol in dealing with catheter-related infections is the removal of the catheter, although this is not always feasible with a port system. The results of our study are in full agreement with this approach, as eradication of all biofilm cells is difficult to achieve.

Consistent with previous studies, limited activity of vancomycin against the *in vitro S*. *epidermidis* biofilms was observed in the present study [28]. Owing to the evidence of development of resistance to vancomycin reported [29], daptomycin is recommended as an antimicrobial alternative for the treatment of biofilm associated infections in catheterized patients [4].

Mascio et al. showed that daptomycin at higher concentrations (100 μg/mL) has bactericidal activity against stationary-phase *S*. *aureus* cells and concluded that this unique ability of daptomycin has a direct application on biofilm-associated cells [30].

Our results showing very promising therapeutic activity of high dose daptomycin (160 μg/mL) against *S*. *epidermidis* are in agreement with a recent study using daptomycin-lock therapy in a rabbit catheter model [31].

Analysis of the location of FISH-positive cells in the biofilms on antibiotic-treated catheters showed that daptomycin penetrated throughout the depth of the biofilm. In keeping with the findings of Stewart et al. the results obtained clearly indicate penetrance of the antibiotic is not a limiting factor in the treatment of *S*. *epidermidis* biofilms with daptomycin [32].

Very large-scale studies would be needed in order to prove the efficacy of antibiofilm agents and would involve a considerable length of time before the beneficial effects begin to reach the patient. Therefore, it is imperative to employ *in vitro* testing of antimicrobials. FISH is a relevant method to test not only anti-biofilm agents but also infected catheters from patients.

## Supporting information

S1 Fig Schematic illustration of the *in vitro* biofilm model for growing *S*. *epidermidis* biofilms. Each biofilm reactor consisted of four polyurethane catheters (Instech Solomon 3 Fr BPU-T30, length 3 cm, outer diameter 0.91 mm, inner diameter 0.58 mm) and an air filter (pore size 0,22 μm, Carl Roth GmbH, Germany), all catheters were connected via tubes to a peristaltic pump (Ismatec^®^ Reglo Digital) for pumping of antibiotic and control solutions. The numbers shown the diagram represent: (1) biofilm reactor with bacterial suspension, (2) air filter, (3) polyurethane catheter, (4) reservoir with medium with or without antibiotic solution and (5) peristaltic pump.
(TIF)

S2 Fig Illustration showing the processing of the catheter samples in methacrylate. Each catheter is cut into four equal sections. These are upright embedded into methacrylate resin. The methacrylate block is sectioned into a total of 32 cross sections per catheter.
(TIF)
