# Supplementary figures and images for: Effect of daptomycin and vancomycin on Staphylococcus epidermidis biofilms: An in vitro assessment using fluorescence in situ hybridization
Source: PLoS One. 2019 Aug 27;14(8):e0221786. doi: 10.1371/journal.pone.0221786 (PMC6711592; doi:10.1371/journal.pone.0221786)

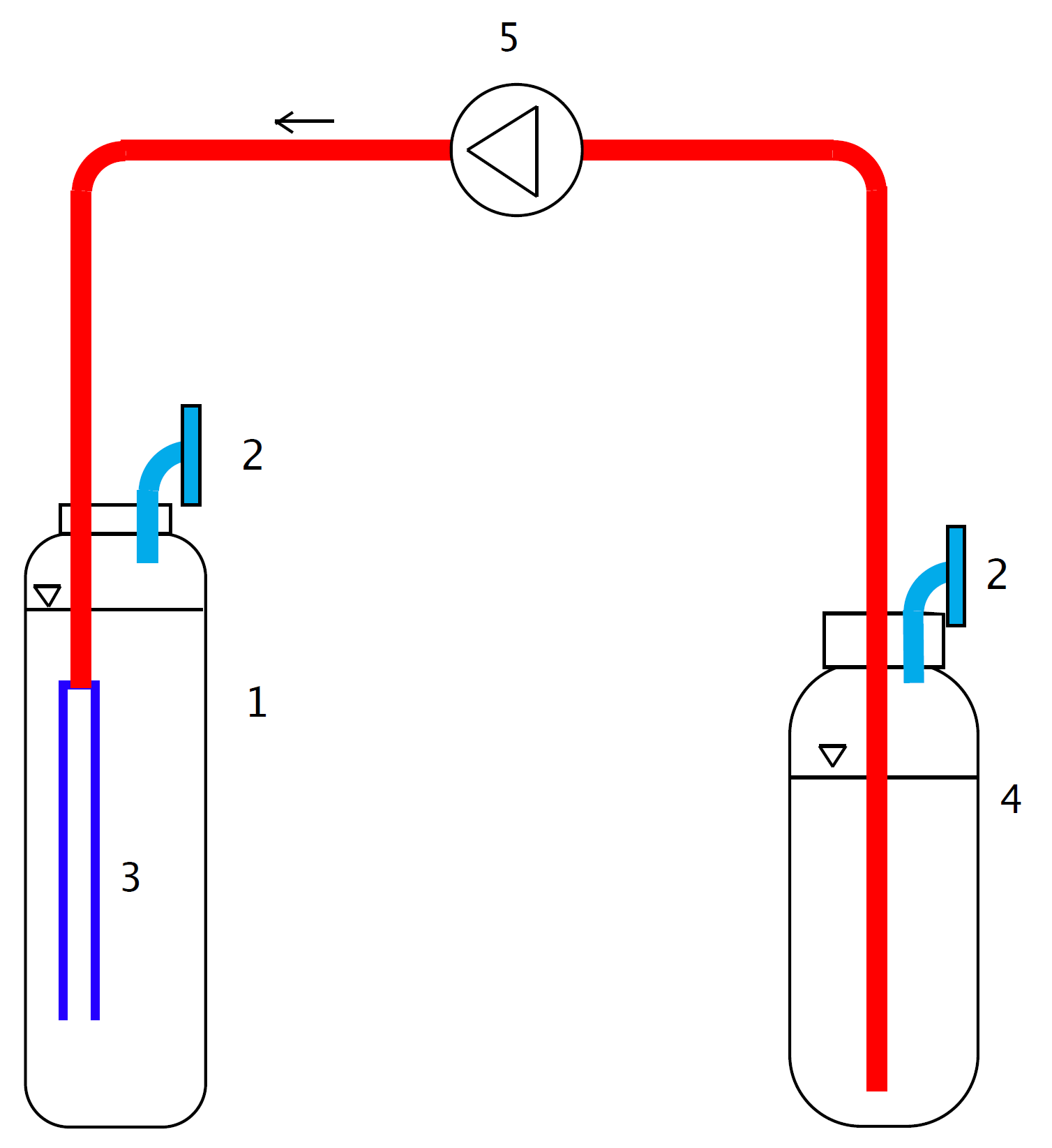

Supplement: S1 Fig — Each biofilm reactor consisted of four polyurethane catheters (Instech Solomon 3 Fr BPU-T30, length 3 cm, outer diameter 0.91 mm, inner diameter 0.58 mm) and an air filter (pore size 0,22 μm, Carl Roth GmbH, Germany), all catheters were connected via tubes to a peristaltic pump (Ismatec® Reglo Digital) for pumping of antibiotic and control solutions. The numbers shown the diagram represent: (1) biofilm reactor with bacterial suspension, (2) air filter, (3) polyurethane catheter, (4) reservoir with medium with or without antibiotic solution and (5) peristaltic pump. (TIF) [file pone.0221786.s001.tif]

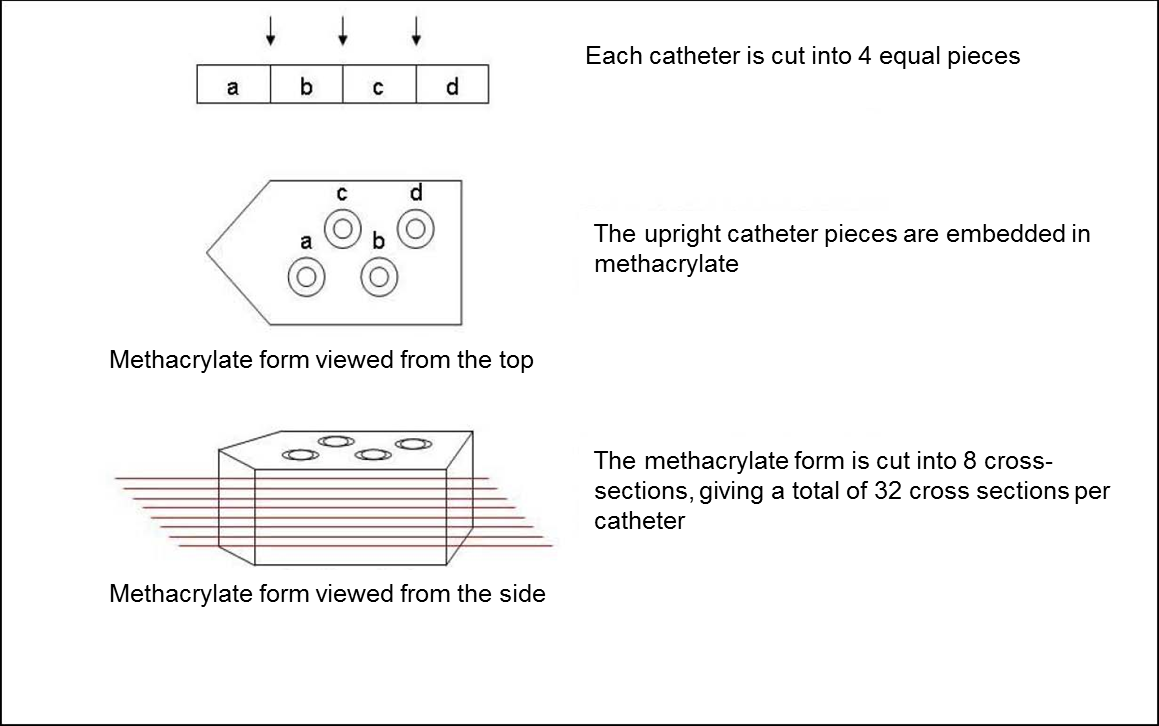

Supplement: S2 Fig — Each catheter is cut into four equal sections. These are upright embedded into methacrylate resin. The methacrylate block is sectioned into a total of 32 cross sections per catheter. (TIF) [file pone.0221786.s002.tif]
